# Hand Injuries in the Polish Silesian Paediatric Population—An Exploratory Cross-Sectional Study of Post-Traumatic X-rays

**DOI:** 10.3390/medicina56100550

**Published:** 2020-10-20

**Authors:** Maciej Cebula, Sandra Modlińska, Magdalena Machnikowska-Sokołowska, Jacek Komenda, Agnieszka Cebula, Jan Baron, Katarzyna Gruszczyńska

**Affiliations:** 1 Department of Radiodiagnostics and Invasive Radiology, School of Medicine in Katowice, Medical University of Silesia, 40-752 Katowice, Poland; smodlinska@sum.edu.pl (S.M.); jkomenda@sum.edu.pl (J.K.); janb@onet.pl (J.B.); 2 Department of Diagnostic Imaging, School of Medicine in Katowice, Medical University of Silesia, 40-752 Katowice, Poland; magdams@onet.pl (M.M.-S.); kgruszczynska@poczta.onet.pl (K.G.); 3 Department of Pediatric Neurology, Medical University of Silesia, 40-752 Katowice, Poland; cebula.ap@gmail.com

**Keywords:** emergency medicine, fracture, hand, paediatrics, radiology

## Abstract

*Background and objectives:* In the paediatric population, hand injuries are one of the most frequent injuries and the second most frequent area of fracture. It is estimated that hand injuries account for up to 23% of the trauma-related causes of emergency department visits. Not only are they a significant factor in health care costs, but they may also lead to detrimental and long-term consequences for the patient. The discrepancy observed between the published studies suggests a geographical variation in their epidemiology. The aim of this study is to determine the localisation of injuries and fractures involving the hand in the paediatric population of the Polish Silesia region. This exploratory cross-sectional study involved 1441 post-traumatic hand X-ray examinations performed at the Department of Diagnostic Imaging of the John Paul II Upper Silesian Child Health Centre in Katowice between January and December 2014. *Materials and Methods:* The study group consisted of 656 girls and 785 boys who were 11.65 ± 3.50 and 11.51 ± 3.98 years old, respectively (range: 1–18 years). All examinations were evaluated for the location of the injury and presence of fracture(s). *Results:* Finger injuries were dominant (*n* = 1346), with the fifth finger being the most frequently injured (*n* = 381). The majority of injuries were observed among children who were 11 years old (*n* = 176), with a visible peak in the 11- to 13-year-old group. A total of 625 bone fractures were detected. Fractures of the proximal phalanges (*n* = 213) and middle phalanges (*n* = 159) were most common, and fifth finger (*n* = 189) predominance was again observed. A gender-independent positive correlation was found between patients’ age and finger injuries (*p* < 0.01) as well as metacarpal injuries (*p* < 0.01). There was no correlation between patients’ age and fractures in these locations (*p* > 0.05). Metacarpal injuries (*p* < 0.01), finger injuries (*p* < 0.01), fractures (*p* = 0.01), and fractures with displacement (*p* = 0.03) were more common among males regardless of age. *Conclusions:* The results indicate that 11-year-old boys are at an increased risk of hand injuries and fractures. The distal and middle phalanges of the right hand, especially of the fifth digit, were the most susceptible to fracture localisation. Thus, injuries in these areas should be perceived as most likely to cause fractures and therefore demand careful examination.

## 1. Introduction

Hand injuries are one of the most common injuries among children [1]. They are related not only to children’s stress and possible impairment but are also an important economic problem. These injuries comprise about 13.0–23.0% of injury-related and 2.3% of general emergency room visits [2,3,4,5]. The hand is the second most common site of fractures (after the forearm) in most studies, but exceptions to this rule have been reported [1,6].

An approximate projected annual incidence rate of hand injuries in children ages 0–16 years in the UK is estimated at 418/100,000 [3]. Time to diagnosis is important, as fractures treated surgically within a week have better outcomes [5]. Misdiagnosis has been shown to occur at a rate of up to 8% [7].

The socioeconomic costs of hand injuries result from multiple factors: parents’ absence from work; costs of diagnosis, treatment, and hospital stays; the child’s impaired working ability; and more. The annual health care costs of hand injuries in children ages 0 to 6 years from three catchment areas in south Sweden ranged from EUR 398,762 in 1996 to EUR 247,540 in 2000, and the average number of patients per year was 86 [2]. Another study from the USA reported that emergency department charges for paediatric upper limb injuries from 2008 to 2012 were USD 21.2 billion as a result of 11.7 million visits [8].

The literature regarding the epidemiology of hand injuries in the eastern European paediatric population is scarce. The discrepancy between the published data suggests a geographical distribution of their quantity and localisation [6,9,10,11,12], and thus such an analysis is needed for this specific region.

Knowledge of the most frequent localisation of fractures in specific paediatric populations ensures not only faster diagnosis, better results and lower health care costs but could also lead to the development of strategies to decrease the incidence of hand injuries. The purpose of this study was to present reliable data on hand injuries and fractures among Silesian children with special regard to identifying the most affected age, gender and susceptible localisation. The goal is to help health care providers to identify high-risk patients and shorten the admission to diagnosis and treatment times.

## 2. Materials and Methods

This exploratory cross-sectional study involved data from the charts of 1441 post-traumatic hand X-ray examinations performed from January to December of 2014 at the Department of Diagnostic Imaging of the John Paul II Upper Silesian Child Health Centre in Katowice. This medical centre provides medical care for the paediatric population from newborns to 18 years old and offers both out-patient and in-patient care. It is one of the largest paediatric hospital emergency departments operating in the Upper Silesian region, serving a population of around 760,000 children in 2015. It provides around 17,000 emergency medical services per year. Due to its multi-profile character, it is a preferred hospital for all types of traumatic injuries in children in the region.

We obtained all hand X-rays performed in 2014 for the purposes of this study. The analysis covered injuries of the phalanges and metacarpal bones. A local ethics committee, the Komisja Bioetyczna Śląskiego Uniwersytetu Medycznego w Katowicach, waived the requirement to obtain ethical approval for this study. Patient records were obtained in the form of an anonymised report compiled from the hospital’s internal database, and identification of individual patients solely on the collected data was impossible.

The time and anatomical localisation of the injury, presence of the fracture, and patient’s age and gender were noted. The number of injuries and fractures and their proportion were the main outcome variables; gender, age, and the month the injury was sustained were used as independent variables for stratification. Using Rajesh et al.’s [9] age categorisation, patients were divided into five age groups: 0–4 years, 5–8 years, 9–12 years, 13–16 years, and 17–18 years. We categorised fractures involving the growth plate in accordance with the Salter–Harris classification.

The study group consisted of 656 girls and 785 boys who were 11.65 ± 3.50 and 11.51 ± 3.98 years old, respectively (range: 0–18 years). Table 1 shows the number of injuries and fractures by localisation for the entire study group and distributed by gender.

Statistical analysis of the gathered data was performed using the Statistica 12.0 software (StatSoft, Kraków, Poland). To evaluate the normality of distribution, the Shapiro–Wilk test was used—none of the variables showed normal distribution. To compare the differences in quantitative variables between two groups, the Mann–Whitney U test was used. To compare between more than two groups, the Kruskal–Wallis test was applied. For all the tests, α = 0.05 was considered significant.

## 3. Results

**Figure 1:**
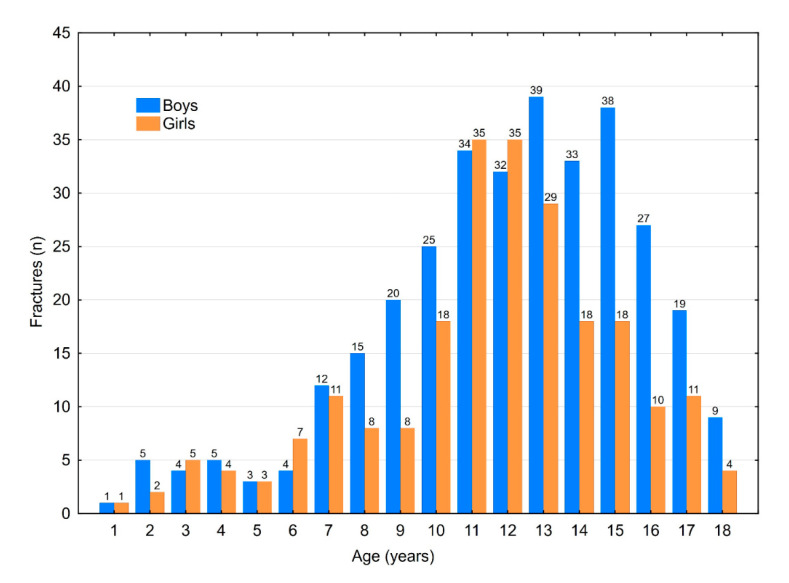
Number of fractures distributed by age and gender.

**Figure 2:**
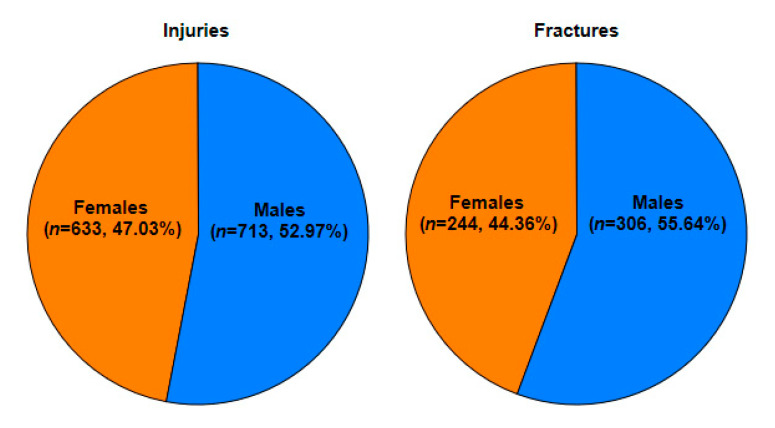
Ratio of finger injuries to fractures distributed by gender.

There was no significant difference between genders in regard to injuries. In the group with fractures, there was a significant difference in age between genders (*p* = 0.029), with older males more likely to sustain fractures (Figure 1). We observed a higher injury-to-fracture ratio in males (*p* = 0.008) (Figure 2).

**Figure 3:**
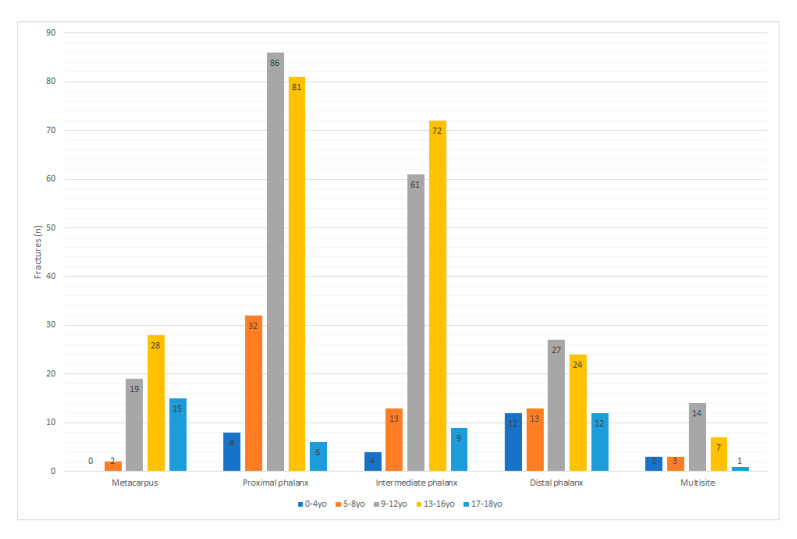
Localisation of fractures distributed by age group.

No significant difference in injury-to-fracture ratio was observed in regards to age group (*p* = 0.140), side (*p* = 0.435) or the month in which the injury occurred (*p* = 0.757). The distribution of fractures by age group and gender is presented in Table 2. A significant difference was observed in terms of the age group and fracture localisation (*p* < 0.001) (Figure 3).

Significant differences were found in the number of fractures between metacarpus and proximal phalanx as well as metacarpus and distal phalanx between age groups.

**Figure 4:**
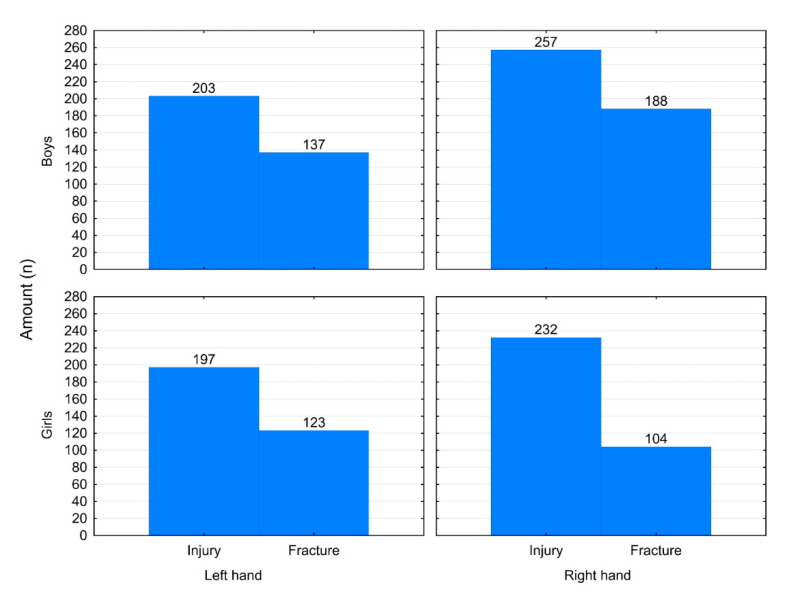
Left-hand vs. right-hand injuries and fractures distributed by gender.

There was a significant difference between males and females regarding which hand (right or left) was injured or fractured (*p* = 0.038 and *p* = 0.005, respectively). Males had a predilection to injuring the right hand, while in females difference was not significant (Figure 4).

**Figure 5:**
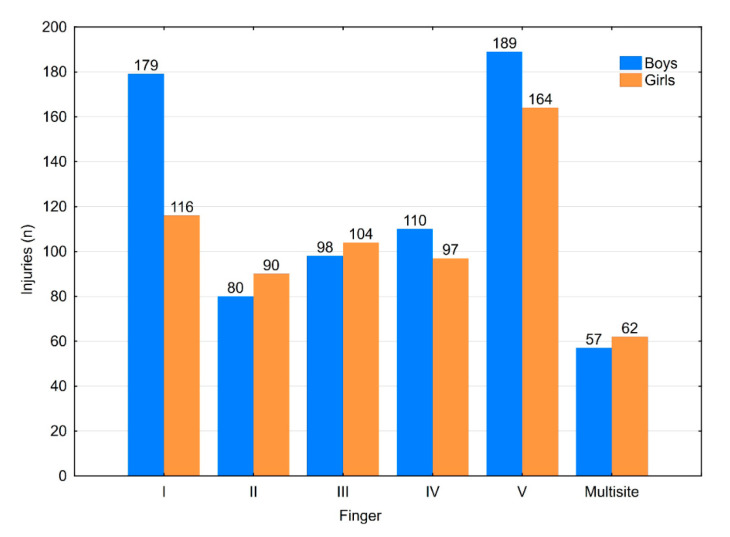
Injury localisation distributed by gender.

The distribution of injuries by finger showed significant differences between genders (*p* = 0.031). However, no significant difference was observed between genders for the distribution of fractures by finger (*p* = 0.594) (Figure 5). There were significant differences between the age groups regarding which finger(s) were injured (*p* = 0.010) (Table 3).

**Figure 6:**
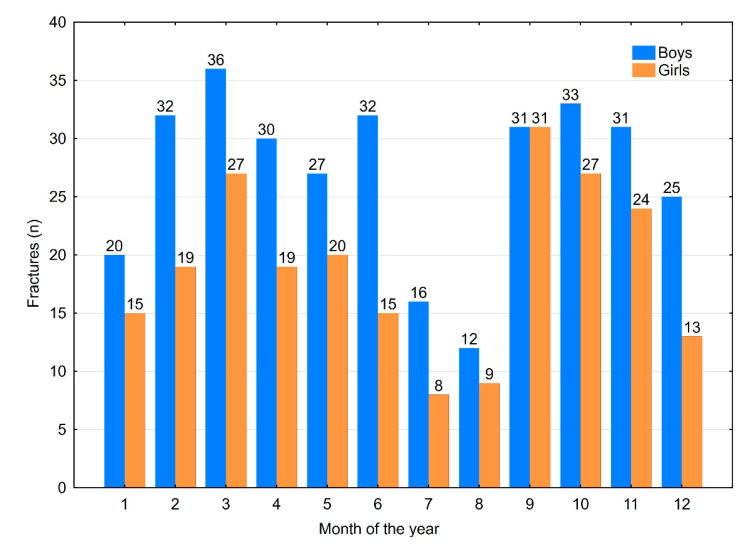
Monthly distribution of fractures allocated for gender.

**Figure 7:**
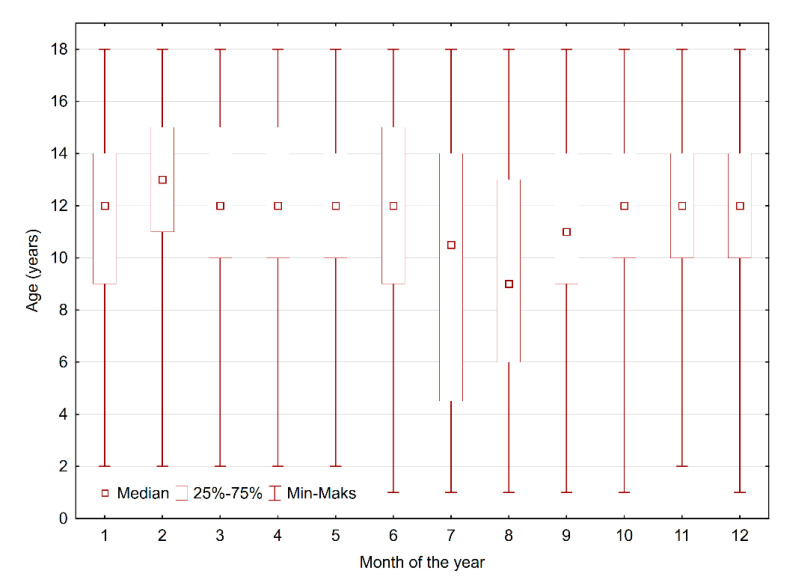
Monthly distribution of injuries allocated for age.

The distribution of fractures by month of occurrence allocated for gender is presented in Figure 6. No significant difference in the month of occurrence was observed for hand injury (*p* = 0.912) or fracture (*p* = 0.865) based on localisation. For the injury group, a difference in patient age was only observed for one month—August—when a significantly younger group was observed (*p* < 0.001). No significant differences in the month of occurrence were observed in the fracture group when allocated for age (*p* = 0.484) (Figure 7).

**Figure 8:**
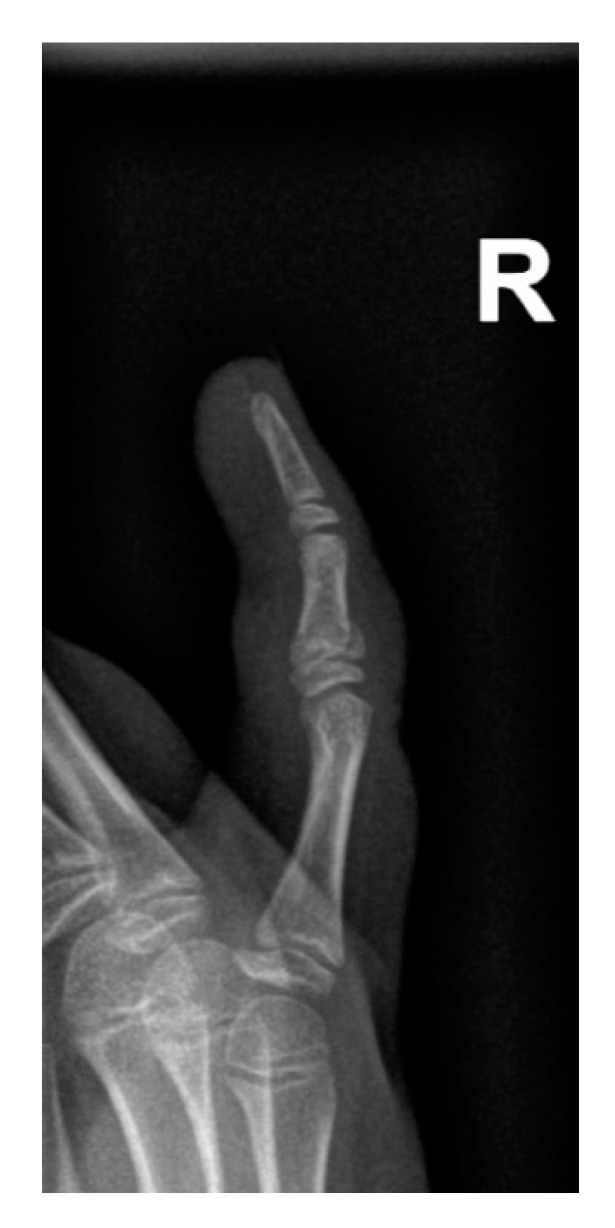
Example of a Salter–Harris fracture of the intermediate phalanx of the fifth finger on the right hand.

Only 21 Salter–Harris fractures were observed, accounting for 1.46% of all injuries and 3.36% of all fractures, with type II being dominant (Table 4). Due to the low number of cases, performing a reliable statistical analysis was impossible. An example of a Salter–Harris fracture is presented in Figure 8.

## 4. Discussion

The epidemiology of hand fractures may vary between different geographical areas, and thus analyses for specific populations of children are needed [1,6,7]. These reported differences may arise from ethnic and cultural differences, environmental factors resulting in different activities and different study methodologies.

Nearly 40% of the X-rays examined in the current study confirmed fractures. This percentage was similar to the findings of studies on sport-related injuries [13] and more than double the results of other studies showing a fracture prevalence of 15–16% [9,13,14]. One study on metacarpal and finger fractures in the Singaporean paediatric population found a misdiagnosis rate of 8% [7]. Of 16 misdiagnosed patients, 11 were over-diagnosed and three were under-diagnosed in terms of the number of fractures. It was impossible to assess how many patients with fractures were overlooked [7]. Due to the lack of follow-up information, we were unable to compare the misdiagnosis results of that study with our own.

The gender differences in the number of hand injuries and fractures in our study is similar to the results of other studies in the literature [3,5,6,7,9,14,15,16,17]. The predominance of fractures among males increases with age and is most apparent among teenagers [3,5,6,12]. The childhood risk of sustaining a fracture between 0 and 16 years is around 50% higher among boys (12–64% for boys and 6–40% for girls) [1,13]. This difference is partially explained by Stracialli et al.’s [13] recent study of sport-related injuries in the paediatric population. Based on an analysis of 2133 cases of youth injuries, the researchers concluded that overuse injuries are more common among female athletes (62.5%) and trauma injuries among male athletes (58.2%), with fractures occurring nearly twice as often among boys. In addition, injuries in boys are more likely to occur in the upper limbs (29.8% of all injuries in boys vs. 15.1% of all injuries in girls). The reasons behind these differences involve distinct movement patterns between genders, anatomical and functional disparities, and the higher ratio of males in contact/collision and risk-related sports [13]. In older children, the mechanism of injury is most often related to sport [16]. However, contrary to other reports, one study found no statistical difference in the number of injuries between girls and boys ages 12–16 years [14]. The predominance of male sex for both hand injuries and fractures was also observed in our study.

In the present study, both injuries and fractures increased after the age of 7 years, with a peak at 11–12 years and slight decline afterwards. This finding is in agreement with other studies [1,15,17]. This age-dependent peak is most likely related to an increase in sport activities among school-age children as well as a high rate of fights among peers [1,7,13]. In some studies, a more bimodal distribution of age was found, with an additional peak at ages 0–2 [3,14]. Studies from Singapore, the UK and the USA reported varying results, with a rise in the incidence of hand fractures among children ages 10 to 11 and a peak at ages 14 to 15 [3,7].

In this study, 21 cases of growth palate fractures (1.46% of all fractures) were observed. This percent is much lower than the findings of other studies, with Bhende [16] reporting growth palate fractures in 32.6% of cases and Mahabir [17] in 38.8%. Due to possible disruption of further growth and the necessity of surgical treatment, this particular kind of fracture has important implications, but it is also the most commonly misdiagnosed [7,16,17].

Localisation of fractures in relation to age differs between studies. The study by Rajesh et al. in the UK found that fractures most commonly occurred in the distal tuft in children ages 0–4, the distal phalanx in ages 5–8, the proximal phalanx in ages 9–12, and the neck of fifth metacarpal in ages 13–16 [9]. Other studies have confirmed this trend that older children tend to have more proximal fractures [3,4,7]. This emerging pattern may be explained by different mechanisms of injury at different ages; younger children usually suffer from crushing (e.g., finger closed in a door), while older children sustain trauma related to sport and fights [2,3,14,16].

However, a Canadian study found that metacarpal injuries were dominant in the youngest group (0–3 years), constituting 48% of all fractures in this group, while older children sustained injuries mainly to the phalanges [15]. In our study, metacarpal fractures were dominant among 17 and 18 year olds.

In this study, the most common location of fracture was the fifth finger and proximal phalanx, which is agreement with some studies done in other regions [7,15]. However, other studies found that the fifth metacarpal was the most fractured [3,9,16,17]. Additionally, a study in the US state of Florida found that the thumb was the finger most frequently injured [14]. That being said, the majority of studies agree that fractures are most common in the fifth finger and the metacarpophalangeal joint [3,7,9,15,16,17]. In addition, despite the majority of the population being right handed, both hands were equally involved in injuries in females, whereas right-hand injury was more common in males, in agreement with other studies’ findings [3,7,15,16].

The results of our study also concurred with those of another study done in the general population regardless of age. In that study, males between the ages of 16 and 35 were the most likely to sustain hand fractures, the fifth ray was the most often fractured, and the left and right hands were injured in equal amount [17]. One important difference that may explain some of the discrepancies with previously mentioned studies is that in older youth, the most frequently fractured bone is the fifth metacarpal followed by the thumb, with the ring and middle finger also often sustaining injuries. These findings are likely due to the common occurrence of young adult males to get into fights involving punching, leading to the well-known “boxer’s fracture” of the fifth metacarpal. In one study of youth ages 13 to 16, 25.6% of hand fractures among males were a result of punching compared to only 1.8% in females [17].

We also noted some seasonal changes in hand injury frequency, with peaks in March and September and a clear decrease during the summer holidays in July and August. The data on this finding are limited because this study was conducted in a single location that is not near any winter sport centres, thus explaining the low numbers of winter sports-related injuries (such as skiing and snowboarding) in our group.

The literature on seasonal differences in the number of hand injuries is scarce; instead, some authors have concentrated on the time of day or did not collect data for an entire year [14,15]. Similar to our study, a biphasic seasonal tendency—with a decrease during the summer months and slight increases in September and March—was observed in a Dutch study of the general population [11]. One possible explanation for this seasonal tendency is holiday travel. This finding contradicts those of other studies showing that most injuries take place during leisure and outside-school time, such as holidays, due to higher participation in sport activities and more time spent outside [14,15]. For example, in a study in Turkey and another in the USA, the lowest incidence of hand injuries was observed in the winter and early spring, and the highest was around June to September [8,12]. No seasonality was found in a fracture study from Puerto Rico [6].

The current study has some important limitations. We did not have any information on the mechanisms leading to the injury or the patient’s dominant hand. All data were collected from a single paediatric centre. Thus, even though a large number of cases were analysed, the study group may not be representative of the entire population.

## 5. Conclusions

In the Polish Silesian paediatric population, the risk of fractures is highest among male teenagers, with a peak at 11 years old. The fifth finger is the most likely to be fractured, usually at the proximal phalanx. It is also important to note that both the right and left hands are equally likely to be fractured in females, whereas males have a predilection for injury in the right hand.

## Figures and Tables

**Table 1:** Localisation of injuries and fractures with gender distribution.

Localisation	All (*n* = 1441)	Girls (*n* = 656)	Boys (*n* = 785)
Injuries (*n*)			
Fingers	1346	633	713
I	301	116	184
II	218	114	104
III	298	155	143
IV	300	146	154
V	381	178	203
Metacarpus	138	40	98
I	46	15	31
II	20	7	13
III	25	8	17
IV	14	4	10
V	46	10	36
Fractures (*n*)			
Fingers	550	244	306
I	83	32	51
II	59	31	28
III	107	53	54
IV	112	50	62
V	189	78	111
Metacarpus	75	12	63
I	14	4	10
II	12	1	11
III	9	2	7
IV	7	1	6
V	33	4	29

**Table 2:** Distribution of injuries and fractures by age group and gender.

Age (Years)	Injuries (*n*) [F/M]	%	Fractures (*n*) [F/M]	%
0–4	96 (35/61)	6.66	27 (12/15)	4.89
5–8	172 (73/99)	11.94	63 (29/34)	11.41
9–12	553 (269/284)	38.38	207 (96/111)	37.50
13–16	510 (236/274)	35.39	212 (75/137)	38.41
17–18	110 (43/67)	7.63	43 (15/28)	7.79

F—female, M—male.

**Table 3:** Fracture localisation distributed by age group.

Age (Years)	Localisation (*n*)					Total (*n*)
	Metacarpus	Proximal Phalanx	Intermediate Phalanx	Distal Phalanx	Multisite	
0–4	0	8	4	12	3	27
5–8	2	32	13	13	3	63
9–12	19	86	61	27	14	207
13–16	28	81	72	24	7	212
17–18	15	6	9	12	1	43
Total (*n*)	64	213	159	88	28	552

**Table 4:** Distribution of Salter–Harris fractures by type and gender.

Salter–Harris Type	Cases (*n*) [F/M]	%
I	6 (3/3)	28.57
II	9 (5/4)	42.86
III	4 (0/4)	19.05
IV	2 (1/1)	9.52
V	0 (0/0)	0.00

F—female, M—male.
